# Unusual intracystic papilloma arising from ectopic axillary breast tissue: Case report

**DOI:** 10.1016/j.radcr.2023.06.053

**Published:** 2023-07-22

**Authors:** Sara El Malih, Manar Ezzahi, Meriem Haloua, Layla Tahiri, Amal Akammar, Nizar El Bouardi, Badreeddine Alami, Moulay Youssef Alaoui Lamrani, Mustapha Maaroufi, Meryem Boubbou

**Affiliations:** a Department of Radiology Mother and Child, CHU Hassan II Fez, Sidi Mohammed Ben Abdellah University, Fez, Morocco; b Department of Radiology and Interventional Imaging, CHU Hassan II Fez, Sidi Mohammed Ben Abdellah University, Fez, Morocco; c Department of Pathology, CHU Hassan II Fez, Sidi Mohammed Ben Abdellah University, Fez, Morocco

**Keywords:** Accessory breast tissue, Axillary, Papilloma

## Abstract

Accessory breast tissue (supernumerary breast tissue) is due to the absence of regression of the primitive milk lines during embryonic life which extends from the axilla to the groin. It is mostly located in the axilla where it is often confused with the axillary extension of the breast, or any pathological process occurring in armpits. Ectopic mammary glands should not be misdiagnosed as it can potentially undergo the same pathological processes that occur in a normally located breast including benign or malignant breast tumors. We report the case of an intracystic papilloma arising from left axillary accessory breast tissue in a 63-year-old woman. The principal symptom was pain in a palpable left axillary mass without inflammatory signs. Subsequent imaging and histopathologic examination proved it to be a papillary tumor in ectopic breast tissue.

## Introduction

Mammary gland (breast) development begins as ectodermal thickening during the early embryonic phase, and transforms into breast milk lines, that grow along the sides of the embryo during the sixth week of development, extending from the axillary region to the groin [1].

This milk line normally regresses except for 2 segments in the pectoral region that later form breasts. Failure of this line's regression may occur at any level from the axilla to the groin area, which leads to the development of ectopic breast tissue.

This condition is also known as supernumerary breast, ectopic breast, and polymastia, and it frequently develops along the “milk line” or mammary line [2], [3], [4]. Gender, geographical area, race, and inheritance have all been proven to influence the incidence of accessory breast tissue. The prevalence ranges from 0.22% to 6% of the general population [5].

We present a case of axillary accessory breast papilloma that was clinically diagnosed as a lipoma. We are reporting this unusual occurrence because it is uncommon as a seat in this anatomic site. Furthermore, as seen in our case study, this situation might create a diagnostic challenge to clinicians by mimicking lymphoma or other forms of lymphadenopathy. Our goal was to emphasize the significance of including accessory breast and its accompanying pathology in the differential diagnosis of axillary masses.

## Case presentation

A 63-year-old patient presented with a 4-year history of left axillary swelling that became recently painful. The swelling began modest and gradually grew larger until it reached its current size. Except from the recent onset of pain and discomfort, there was no history of nipple discharge, nor history of general symptoms such as cough, fever, weight loss, nocturnal sweating, or other symptoms. Her previous medical and surgical history revealed a thyroidectomy with histological examination of a noninvasive follicular thyroid neoplasm.

Her general state was stable. The following vital signs were recorded: pulse rate of 70 beats per minute; blood pressure of 110/80 mmHg; respiratory rate of 18 breaths per minute; and temperature of 37°C.

**Fig. 1:**
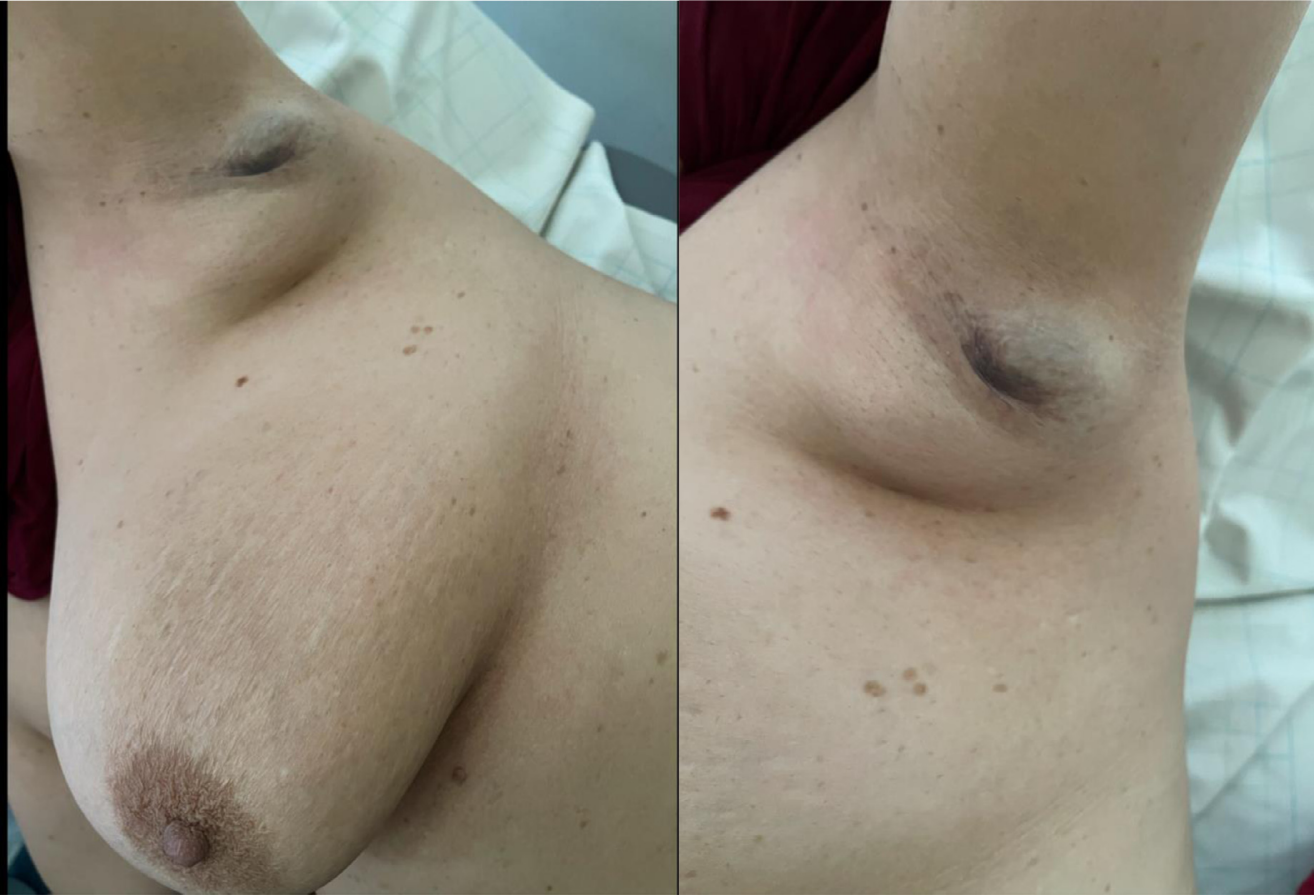
Left supernumerary breast with areola.

There was a 4 × 4 cm solitary mass in the left axilla, well-defined and soft. This lump did not appear to be linked to the skin or the chest wall, and it did not produce any inflammation or skin retraction, however. Skin over the swelling was hyperpigmented suggesting an ectopic areola. There was no abnormality found in the same region on her right breast. The axillary and supraclavicular lymph nodes were absent (Fig. 1).

**Fig. 2:**
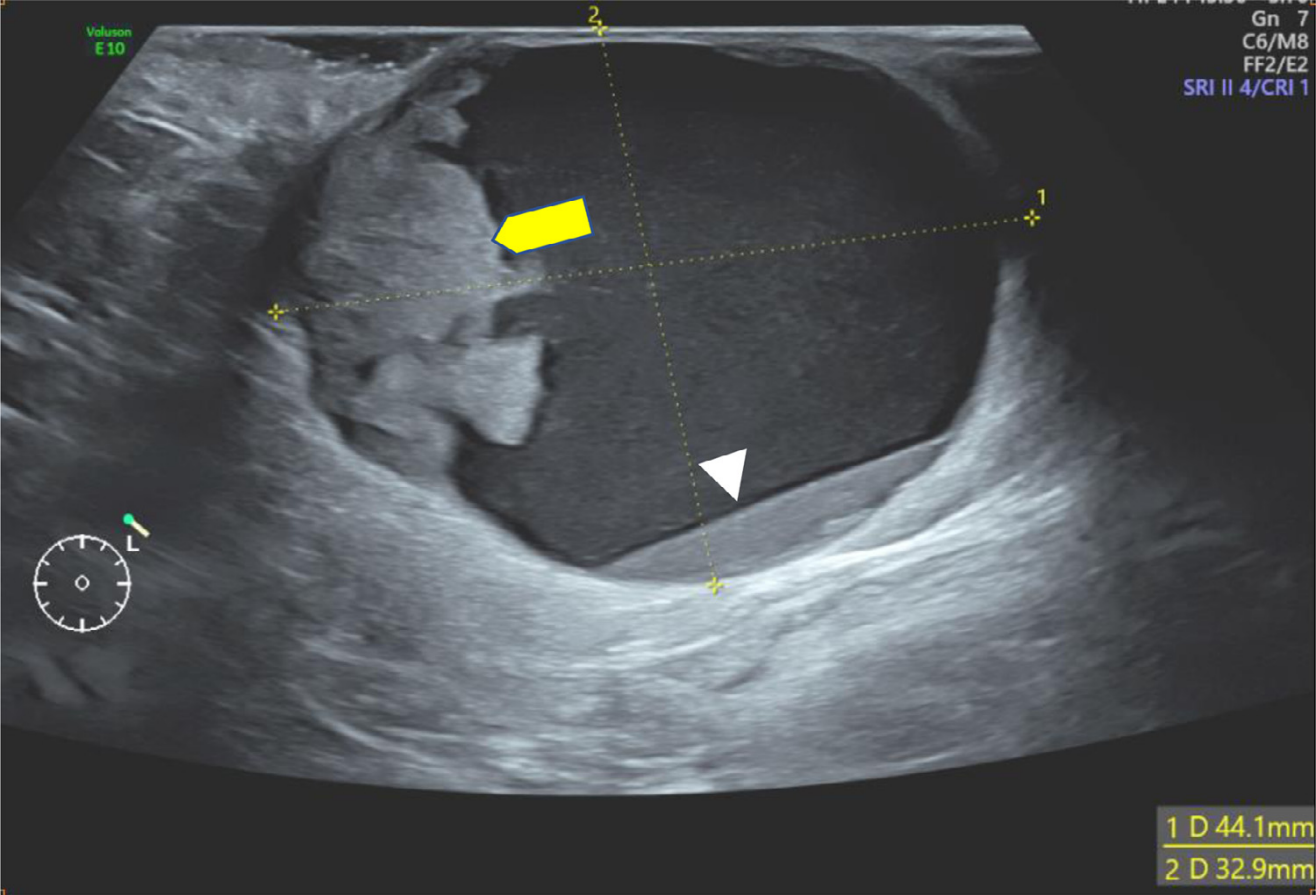
Ultrasound of the left axilla showing a complex cystic and solid mass, containing an echogenic-anechoic liquid level (head arrow), and a tissue component (yellow arrow).

**Fig. 3:**
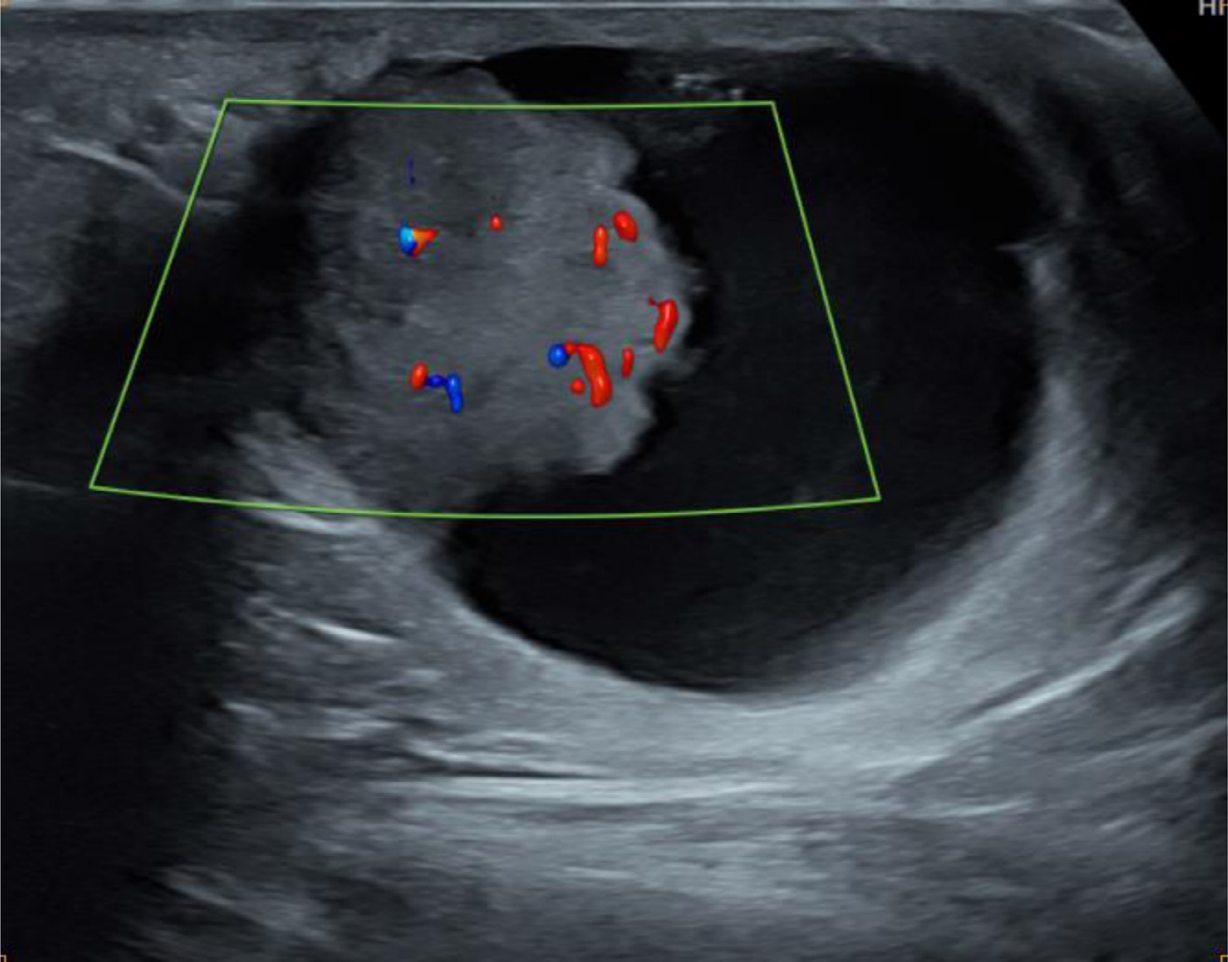
Ultrasound of the left axilla showing Doppler vascularization of the tissue component.

**Fig. 4:**
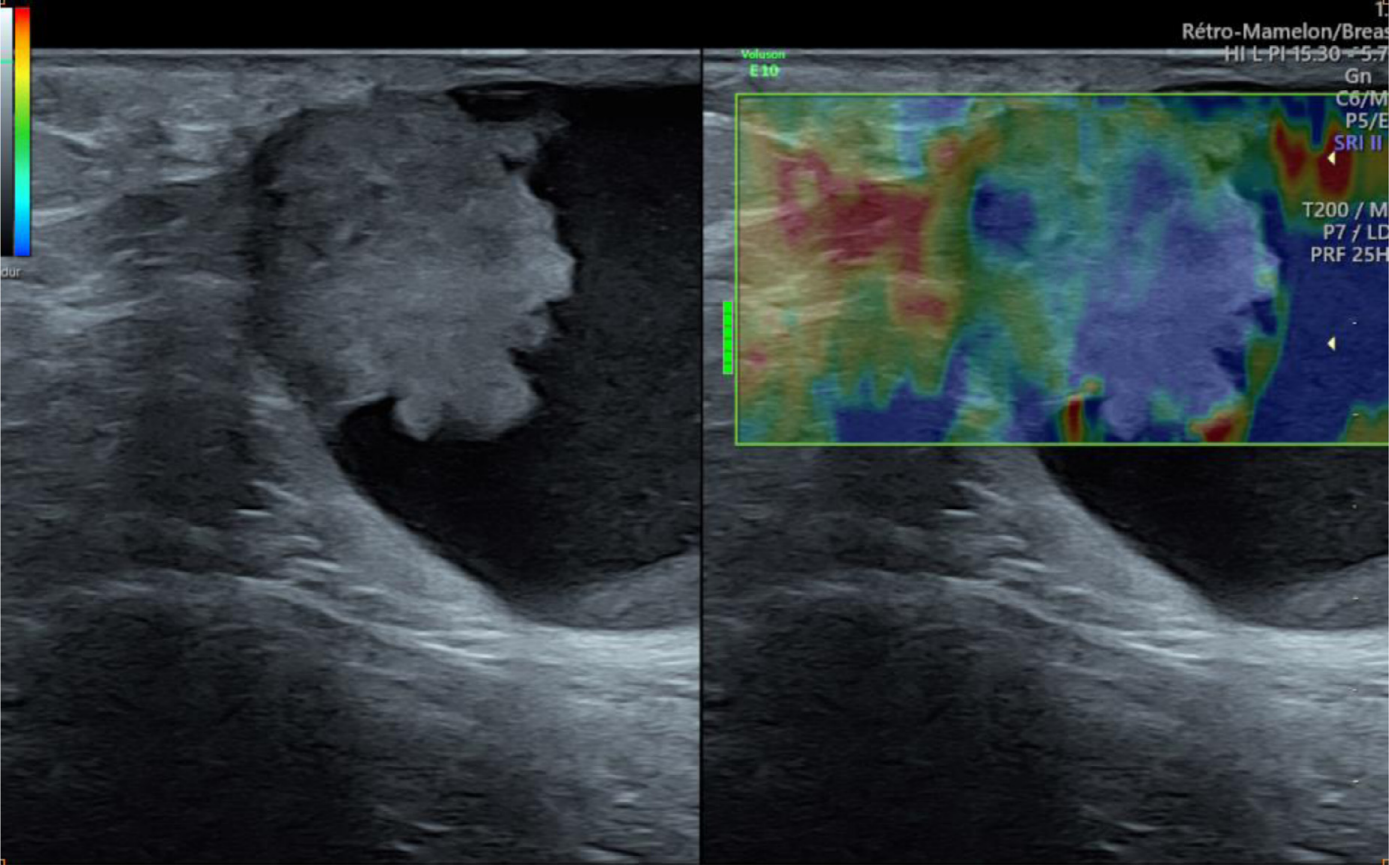
Ultrasound elastography showing hard elasticity of the tissue component.

Ultrasonography was first performed showing a supernumerary breast tissue, of left axillary location, with a complex cystic mass containing an echogenic-anechoic liquid level, and a tissue component, that appears hyperechoic, with ill-defined margins, homogeneous (Fig. 2), with vascular pedicle in color Doppler (Fig. 3) and hard elasticity in ultrasound elastography (Fig. 4).

**Fig. 5:**
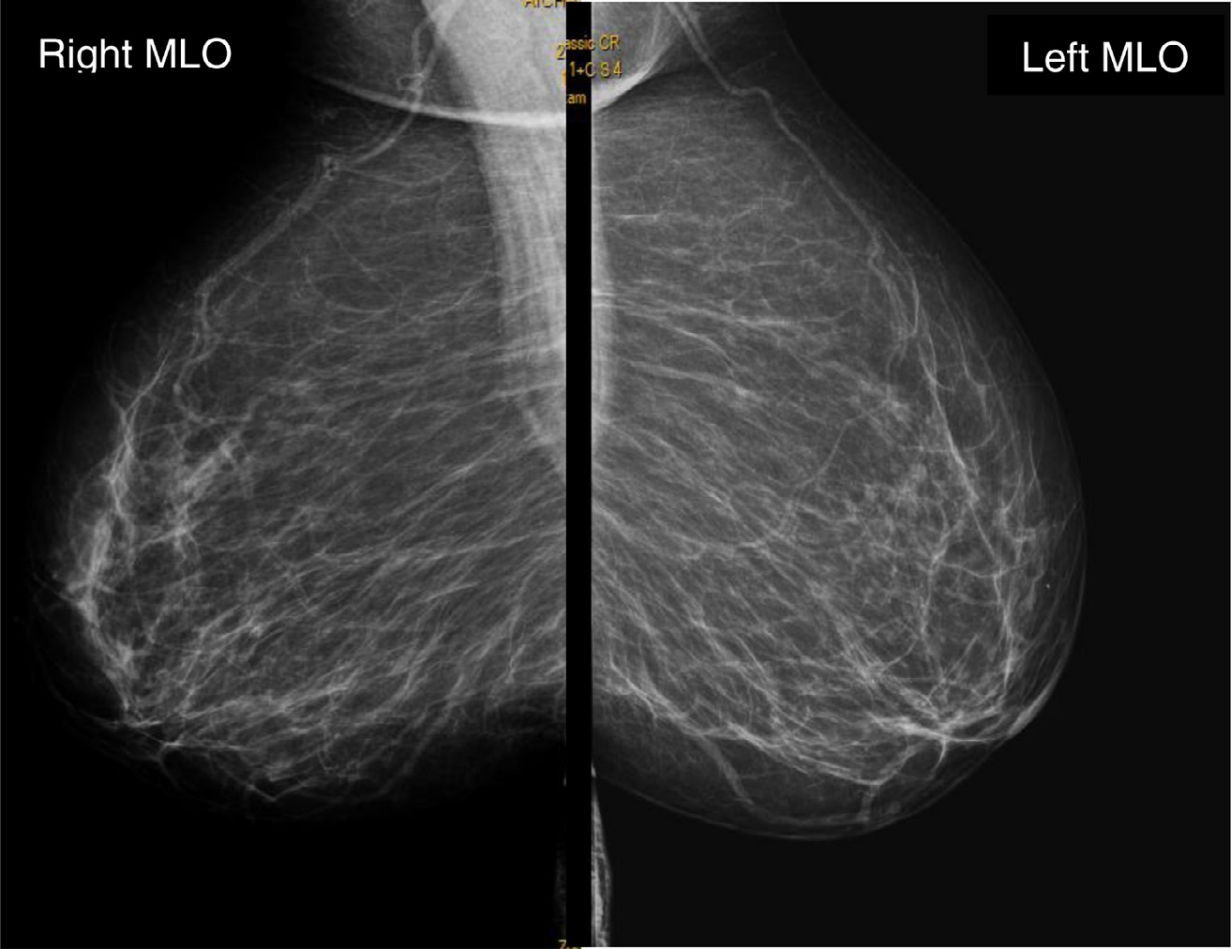
Mammogram in mid-lateral-oblique incidence.

Mammogram and ultrasound of breasts were also performed and showed no notable abnormalities (Fig. 5).

**Fig. 6:**
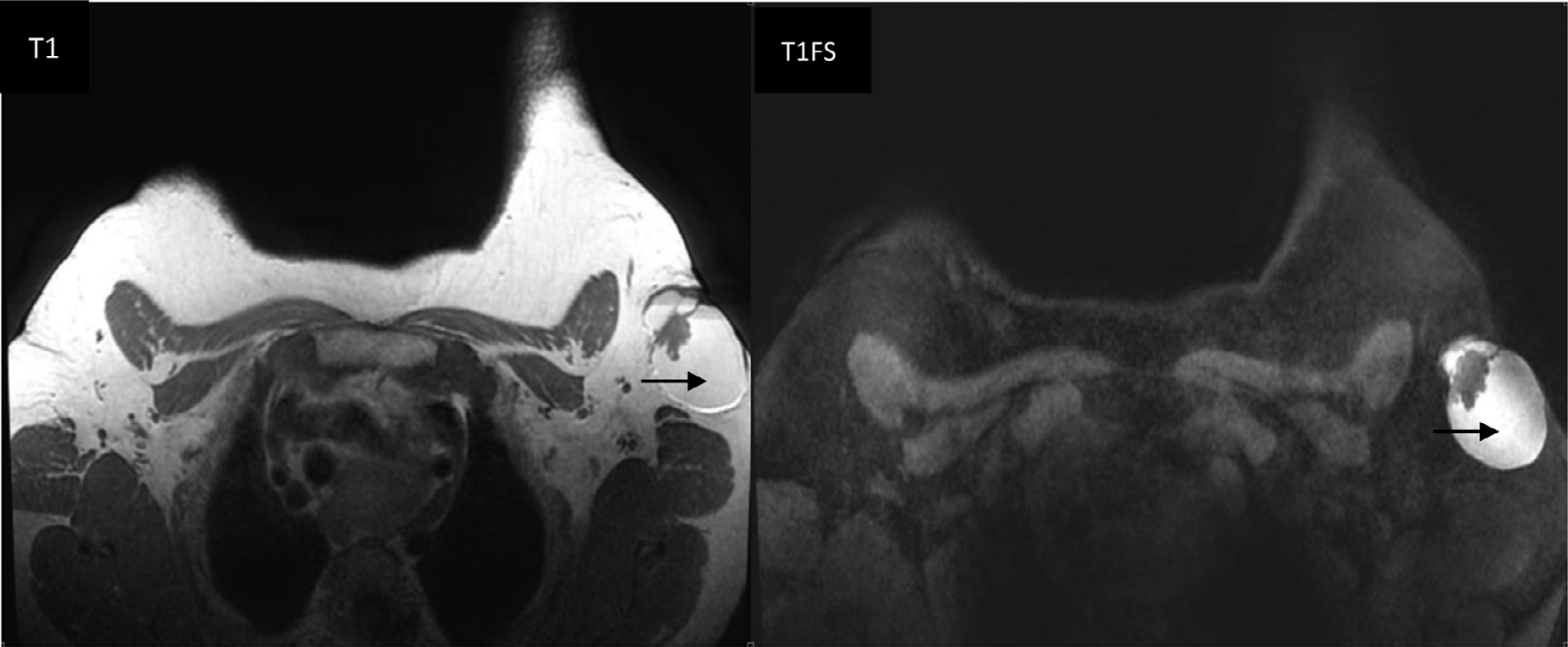
Axial T1-weighted image (left) and axial T1 with fat saturation sequence (right) showing the cystic mass in the left axilla with hemorrhagic content, that appears hyperintense in T1 not suppressed in fat saturation sequence (arrows).

**Fig. 7:**
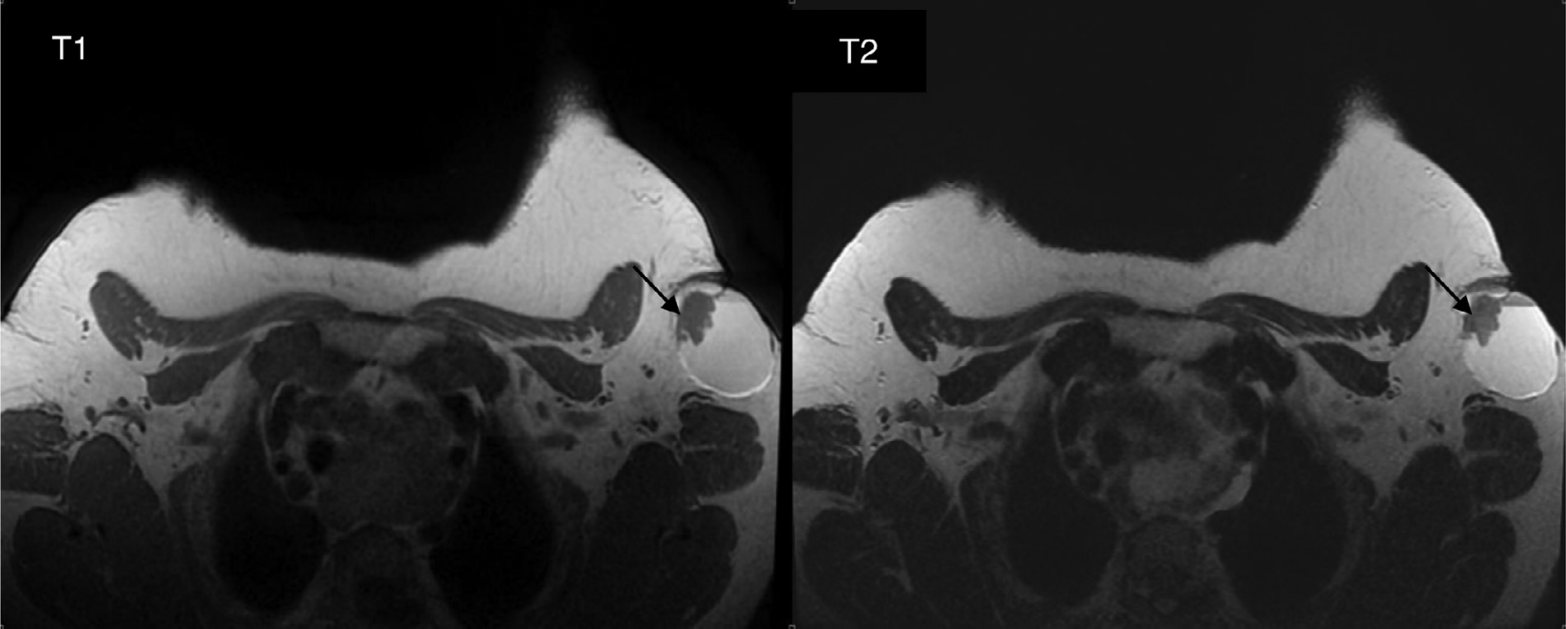
Axial T1- and T2-weighted images show the solid component (arrows) that appears hypointense in T1 with intermediate T2 signal.

**Fig. 8:**
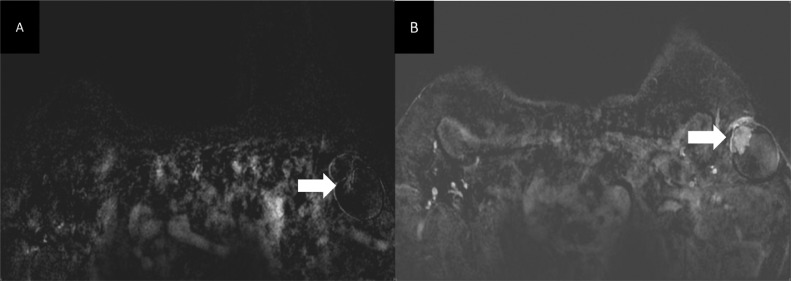
Axial T1 GE 3D subtracted sequences showing rapid homogenous enhancement of the solid component (arrow).

Breast MRI was used to further describe the cystic mass and to seek for other possible breast abnormalities. It revealed a complex mass in the left axilla with hemorrhagic cystic component, and solid content with low signal on T1-weighted images, and intermediate signal on T2-WI. On dynamic sequences after gadolinium, a fast and early enhancement of the solid portion was found (Fig. 6, Fig. 7, Fig. 8).

Ultrasound-guided 16-G core-needle biopsy was performed, revealing an intracystic papilloma. The patient underwent wide local excision for the lesion. She was discharged on the next day, with appointments arranged for follow-up and wound care. Diagnosis was confirmed with the histologic examination of the entire mass.

## Discussion

Accessory breast, also known as polymastia, or supernumerary breasts, is the condition of having more than 2 breasts with or without a nipple and areola. It generally results from an involutional failure of the embryonic milk lines, and may extend from the axilla to the groin but it most commonly arises in the axillary region,

In 1915, Kajava established the accessory breast classification system, which is being used today [[6], [7], [8]] (Table 1). Our case is classified as class III since we only have glandular tissue (detected as papilloma) and areola without nipple.

**Table 1:** Kajava classification [[6], [7], [8]].

Type (class)	Description
Class I	Consists of a complete breast including glandular tissue, nipple, and areola
Class II	Consists of only glandular tissue and nipple, without areola
Class III	Consists of only glandular tissue and areola, without nipple
Class IV	Consists of only glandular tissue
Class V	Consists of only nipple and areola, without glandular tissue (pseudomamma)
Class VI	Consists of only the nipple (polythelia)
Class VII	Consists of only the areola (polythelia areolaris)
Class VIII	Consists of only hair (polythelia pilosa)

Accessory breast tissue responds to hormones and can develop benign and malignant pathologic processes comparable to those found in normal breast tissue, such as fibrocystic disease, intraductal papilloma, lactating adenoma, fibroadenoma, and carcinoma [[9], [10], [11], [12]].

While papilloma is a frequent benign lesion in normal breast tissue, that affects 2%-3% of the population [[13], [14]], its presence in accessory breast tissue is uncommon. Papilloma may arise in a duct as a single mass or multiple masses, or develop into a cystically dilated duct known as intracystic papillomas which was the case of our patient. Unlike intraductal papillomas, intracystic papillomas are seldomly found and tend to occur in postmenopausal women [[15], [16]]. Clinically, papillomas may present with bloody or clear nipple discharge or a palpable sub areolar mass [[17], [18]].

On mammogram, they may present as multiple circular or linear dense soft tissue masses with well-defined or partially circumscribed margi [[19], [20]]. Ultrasound findings describe 3 main patterns of papillomas, including (1) intraductal mass with or without ductal dilatation, (2) intracystic mass due to concomitant excess fluid production in a blocked duct, and (3) primarily solid pattern with intraductal mass completely filling the duct [[21], [22]].

Diagnostic and therapeutic approaches for tumors in accessory breast tissue are the same as for a normal breast mass. Yet, because of its rarity and lack of suspicion, diagnosis may be neglected, delaying prompt treatment.

## Conclusion

While examining a patient with axillary swelling, accessory breast tissue conditions must be addressed as a differential diagnosis for early identification and therapy. This case demonstrates that accessory breast tissue, like normal breast tissue, is subject to the same physiologic and pathologic disease processes, including neoplasms. The majority of care is surgical intervention with excision, when tumors or nodules are discovered along the mammary line, the existence of breast tissue should be investigated to rule out the correct diagnosis and to guide the proper management strategy.

## Ethics approval and consent to participate

Not applicable.

## Availability of data and materials

The data sets are generated on the data system of the CHU Hassan II of Fes, including the biological data and the interventional report.

## Patient consent

I, the author of the article “Unusual intra ductal papilloma in ectopic axillary breast tissue: Case report,” approve that the patient gives her consent for information to be published in *Radiology Case Reports*.
